# Artificial Somnambulism in Pennsylvania

**Published:** 1847-02

**Authors:** 


*Artificial Somnambulism in Pennsylvania.*—Doct. William B. Fahnestock, of Lancaster, Pennsylvania, has published an article on Mesmerism, or as he terms it, "artificial somnambulism," which will stagger the faith of most christians. Can a man be sane who believes such assumptions? We offer a few extracts as follows:

1. That I consider this condition to be identical with somnambulism, differing only in the manner in which it is produced; somnambulism being the natural state, and this the artificial.

2. That this state is independent of magnetism, electricity, galvanism, sympathy, a nervous fluid, or anything of the kind.

3. That subjects are at all times independent of the so-called operator, in every sense of the word, when they are acquainted with the true nature of the state before they enter it.

4. That it is possible for *any persons* to throw themselves into, or out of, the state, even in an instant, when they have been taught how to effect it.

5. That subjects can be taught to throw themselves into this state, a single finger, a hand, an arm, or any part of the body, independent of the rest, by an act of their own will, at any time they may please, and relieve themselves at pleasure!

I have taught hundreds to do so—but it may not always be prudent for them to enter this state independent of an instructer—for *some*, upon entering the condition for the *first time*, become unconscious of all that is passing around them; and if such persons were to throw themselves into it independent of any one, and had not consented or made up their minds before entering it, to hear or to speak to some one, it is most likely that when in it, and spoken to, they would not hear any one; and in all probability would sleep for a longer or shorter time, without doing anything, and when they did awake would remember nothing, and scarcely know they had been in it at all. Or they might get up and wander about, as is sometimes done by natural somnambulists, and unknowingly get into difficulties or meet with some accident, which might not be very agreeable when they awoke. It is, therefore, always better for those who wish to enter it, to place themselves under the care of some one; and he who understands the nature of the state best, and has had the most experience in its management, is the best calculated for this purpose. When they have entered the state frequently, and have had the proper instructions while in it, the case is very different; they are then able to move about with as much certainty and safety as if they were awake.

6. That when in this state they can hear, see, smell, taste, feel, &c., as well as when awake, whenever they are so disposed; or they can hear or not, see or not, smell or not, taste or not, feel or not, contrary to the will of any one, whenever they may think proper.

7. That they cannot be made to do any thing they are not willing to assent to, and are as much themselves as when awake.

The following case of parturition is given:

She, being clairvoyant, then threw her mind to a distance, and while engaged looking at various objects of interest, passed through the rest of her labor without feeling the least pain—even the last and severe contractions of the uterus, gave her no pain whatever. She was delivered of a large male child, about half an hour after she entered the state the second time. After the child was born, and the placenta was about being removed, considerable hemorrhage ensued—but was immediately arrested by frictions with the hand over the region of the uterus, until the contractions were renewed and the placenta expelled. The frictions over the uterus, which were necessary in this case to renew its contractions, gave her no pain whatever. The same, under ordinary circumstances, every physician knows full well, are scarcely to be endured.

Mesmerism advances in mystery; patients are *now* taught, not only to *diagnose* their own disease, but to *remove* them by *willing* that they shall cease! The following extract presents this supremely ridiculous assumption:

The extent to which I have employed somnambulism in diseases has been very considerable; but the state itself, or the mere being in that state, will produce no relief, if the mind of the subject is not made sensible of the fact, and placed upon the disease. This is an important point, and accounts for the many failures which have hitherto occurred. It is always necessary to get the patient to fix his mind upon his disease, and to make a resolution while in the state that it shall cease to exist when he awakes, &c. It is no matter whether the patient does this of his own accord, or by the advice of his instructor, so that it has been done properly. The effect will be the same, and always in proportion to the determination, &c., he may have exercised.

A very happy illustration of this fact took place last winter while I was in the city of Philadelphia. Dr. Childs, of that place, had been endeavoring to effect a cure in the case of a young lady whose foot (I think from some disease), was drawn or thrown completely round, in a direction precisely opposite to the natural condition of that member. He had been operating in the usual method for some time before I visited the city. He attended a course of my *private lectures*, which were delivered in Dr. Noble's parlors, where he stated the young lady's case to me, and requested to know whether I could suggest any thing for her relief. I at once told him that if he would abandon the old method and divert her mind to the foot, and get her to make a resolution while in the state, that the foot should assume and keep a natural position when she awoke, he could effect it in five minutes. On the following day he succeeded in getting her to do so, and brought the young lady round to Dr. Noble's the same evening, perfectly restored. She was then able to keep the foot in its natural position, and during the evening walked and ran about as well as ever, a blessing which she had been unable to exercise for a long time before. I have no doubt that Drs. Childs and Noble will take pleasure in confirming my statement. The case was notorious in that part of the city, and the cure by many was considered scarcely short of a miracle.—*Boston Med. and Surg. Jour.*

